# Anterior Cervical Corpectomy with Fusion versus Anterior Hybrid Fusion Surgery for Patients with Severe Ossification of the Posterior Longitudinal Ligament Involving Three or More Levels: A Retrospective Comparative Study

**DOI:** 10.3390/jcm10225315

**Published:** 2021-11-15

**Authors:** Takashi Hirai, Toshitaka Yoshii, Kenichiro Sakai, Hiroyuki Inose, Masato Yuasa, Tsuyoshi Yamada, Yu Matsukura, Shuta Ushio, Shingo Morishita, Satoru Egawa, Hiroaki Onuma, Yutaka Kobayashi, Kurando Utagawa, Jun Hashimoto, Atsuyuki Kawabata, Tomoyuki Tanaka, Takayuki Motoyoshi, Takuya Takahashi, Motonori Hashimoto, Kentaro Sakaeda, Tsuyoshi Kato, Yoshiyasu Arai, Shigenori Kawabata, Atsushi Okawa

**Affiliations:** 1 Department of Orthopedic Surgery, Tokyo Medical and Dental University, 1-5-45 Yushima, Bunkyo-ku, Tokyo 113-8510, Japan; yoshii.orth@tmd.ac.jp (T.Y.); inose.orth@tmd.ac.jp (H.I.); yuaorth@tmd.ac.jp (M.Y.); yamada.orth@tmd.ac.jp (T.Y.); Matsukura.orth@tmd.ac.jp (Y.M.); ushiorth20@gmail.com (S.U.); morsorth@tmd.ac.jp (S.M.); egawa.orth@tmd.ac.jp (S.E.); onuma.orj@tmd.ac.jp (H.O.); kobayashi.orth@tmd.ac.jp (Y.K.); utag.orth@tmd.ac.jp (K.U.); 0123456789jun@gmail.com (J.H.); 060211ms@gmail.com (A.K.); the.tomo.rrow@gmail.com (T.T.); motoyoshi.orth@tmd.ac.jp (T.M.); tttt841000@gmail.com (T.T.); hmoto95@gmail.com (M.H.); sakaeda.orth@tmd.ac.jp (K.S.); katoorth@gmail.com (T.K.); kawabata.orth@tmd.ac.jp (S.K.); okawa.orth@tmd.ac.jp (A.O.); 2 Department of Orthopedic Surgery, Saitamaken-Saiseikai Kawaguchi General Hospital, 5-11-5 Nishikawaguchu, Kawaguchi City 332-8558, Japan; kenitiro1122@gmail.com (K.S.); arai.orth@gmail.com (Y.A.)

**Keywords:** anterior cervical corpectomy and fusion, hybrid fusion, ossification of the posterior longitudinal ligament, implant failure, graft subsidence, complications, perioperative outcomes, fusion rate, segmental paralysis, mechanical stability

## Abstract

Various studies have found a high incidence of early graft dislodgement after multilevel corpectomy. Although a hybrid fusion technique was developed to resolve implant failure, the hybrid and conventional techniques have not been clearly compared in terms of perioperative complications in patients with severe ossification of the posterior longitudinal ligament (OPLL) involving three or more levels. The purpose of this study was to compare clinical and radiologic outcomes between anterior cervical corpectomy with fusion (ACCF) and anterior hybrid fusion for the treatment of multilevel cervical OPLL. We therefore retrospectively reviewed the clinical and radiologic data of 53 consecutive patients who underwent anterior fusion to treat cervical OPLL: 30 underwent ACCF and 23 underwent anterior hybrid fusion. All patients completed 2 years of follow-ups. Implant migration was defined as subsidence > 3 mm. There were no significant differences in demographics or clinical characteristics between the ACCF and hybrid groups. Early implant failure occurred significantly more frequently in the ACCF group (5 cases, 16.7%) compared with the hybrid group (0 cases, 0%). The fusion rate was 80% in the ACCF group and 100% in the hybrid group. Although both procedures can achieve satisfactory neurologic outcomes for multilevel OPLL patients, hybrid fusion likely provides better biomechanical stability than the conventional ACCF technique.

## 1. Introduction

Ossification of the posterior longitudinal ligament (OPLL) is a heterotopic ossification of spinal ligaments and a unique degenerative spine disease that causes neurologic disorders in middle and old age [1,2]. Although the prevalence of OPLL in Asian countries is reported to range from 1.9% to 4.3% [2], a majority of patients with OPLL seem to have no neurologic symptoms [3]. However, ossified lesions that develop in the spinal canal compress the spinal cord and nerve roots, causing myelopathy or radiculopathy [4]. Surgical treatment should be provided for patients with progressive myelopathy [5,6], whereas conservative treatment is suitable for minimally symptomatic patients [7].

Surgical strategies have evolved as options for treating OPLL based on various studies. Posterior decompression with fusion was demonstrated to provide indirect decompression and stabilize the spinal structure in multiple segments [8]. However, it is also known that sufficient decompression cannot be achieved via a posterior approach in patients with massive OPLL who have kyphosis, and outcomes are relatively poor in posterior decompression with fusion even after eliminating the dynamic factor [9]. Anterior cervical corpectomy and fusion (ACCF) is a key strategy for achieving adequate decompression in patients with cervical OPLL [6]. However, implant failure often occurs in patients treated with anterior cervical corpectomy, and other complications also frequently occur, such as respiratory problems and dysphagia [10]. It was also reported that ACCF results in higher perioperative complication rates compared with anterior cervical discectomy and fusion (ACDF) [11]. Graft dislodgement immediately after ACCF requires emergent salvage surgery; therefore, posterior surgery is widely used by spine surgeons and neurosurgeons even for the treatment of patients with massive ossification or sagittal malalignment [12]. To overcome these issues, an anterior hybrid technique of combined ACCF and ACDF was developed for patients with multiple segmental OPLL [13,14]. This technique allows for more screws to be placed to stabilize the anterior strut, and it is thought to provide better postoperative stability of the fused segments compared with ACCF for patients with multi-level OPLL [15]. However, no investigations have compared traditional ACCF and hybrid ACCF to perform a detailed verification of the structural stability of constructs in these two surgeries, and no studies have focused exclusively on patients with severe OPLL involving three or more segments. Therefore, we conducted this retrospective study to compare the clinical and radiologic outcomes of anterior decompression with traditional ACCF and hybrid ACCF in patients with severe OPLL.

## 2. Materials and Methods

### 2.1. Patients and Methods

This single-center retrospective cohort study was carried out in accordance with the STROBE guidelines [16] and compared ACCF and hybrid fusion for treatment of patients with OPLL involving ≥3 levels. Patients with a history of previous cervical spine surgery or injury were excluded. The study involved consecutive patients in whom anterior surgery was required to treat severe myelopathy due to a compressive lesion involving at least 3 segments, regardless of the duration of symptoms, in our hospital from 2007 to 2018. We previously performed traditional ACCF for all patients until 2011, and thereafter we performed anterior hybrid fusion where possible for patients in whom the vertebral body in the lesion could be preserved. In principle, anterior cervical surgery was performed in patients with OPLL occupying 50% or more of the anteroposterior diameter of the spinal canal. The level to be decompressed was decided based on the neurologic findings and the presence of spinal cord compression. In addition, we performed corpectomy in the levels that had the most compressive OPLL lesion and applied ACDF in the most proximal or distal segments that had relatively small compressive lesions in the anterior hybrid operation.

### 2.2. Operative Technique 

#### 2.2.1. Anterior Cervical Corpectomy with Fusion (ACCF Group)

**Figure 1:**
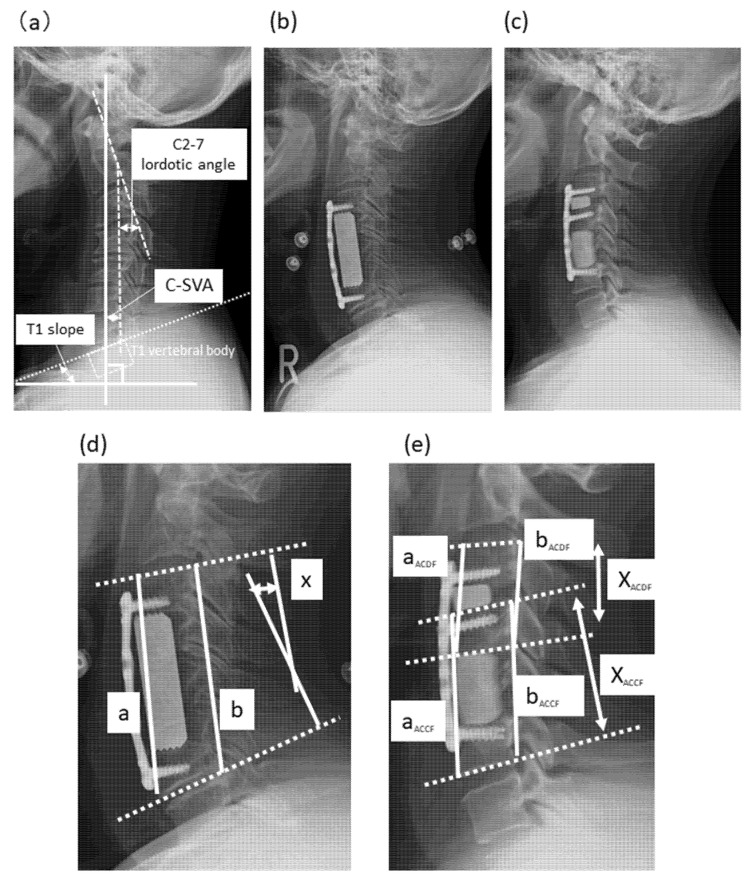
Long semi-constrained plate fixation with artificial bone graft. (**a**) Preoperative radiograph showing the C2–7 lordotic angle, C-SVA, and T1 slope. Postoperative radiographs (**b**) after dual-level corpectomy (C4–5) and ossification floating decompression and (**c**) with dual artificial bone graft after discectomy (C3/4) and single corpectomy (C5). (**d**) Postoperative radiograph showing fused segment angle and fused segment height in the ACCF group. (**e**) Postoperative radiograph showing fused segment angle and fused segment height in the hybrid group.

The operative technique for this procedure was described previously [1]. The anterior decompression with fusion procedure includes partial removal of vertebral body and discs with a strut graft. Segments to be operated were diagnosed based on preoperative radiographic and clinical findings. The length of the bone graft was measured intraoperatively using X-calipers between the upper and lower endplates of vertebral bodies operated in a neutral cervical position. A strut graft collected from the iliac crest or made using artificial bone made from hydroxyapatite (Boneceram^®^; Olympus Corporation, Tokyo, Japan) was used for 2 corpectomies (3 segments), and fibula strut grafts were used for 3 or more corpectomies (4 or more segments). A semi-rigid plate was inserted in all cases. In principle, fixed screws were placed for the distal vertebrae and variable screws for the proximal vertebrae (VENTURE^™^ Anterior Cervical Plate System; Medtronic Sofamor Danek Inc., Memphis, TN; Figure 1a). This technique was performed by five senior spine surgeons. Patients basically wore a neck collar for 2–3 months postoperatively.

#### 2.2.2. Anterior Hybrid Procedure (Corpectomy-Discectomy with Fusion, Hybrid Group) 

The ≥2 levels that caused relatively severe cord compression were treated with corpectomy (as in the ACCF group), and the remaining disc level was treated with discectomy. Autograft or artificial bone graft (Boneceram^®^; Olympus Corporation, Tokyo, Japan) for segments treated with corpectomy and cervical fusion cage or artificial bone graft were placed with a plate and 6-screws fixation (Figure 1b). This technique was performed by four senior spine surgeons. Patients basically wore a neck collar for 2–3 months postoperatively. 

### 2.3. Clinical Evaluations

Most of the patients visited at 3, 6, 12, 18, and 24 months for postoperative clinical and radiologic follow-up. All patients were followed up for 2 years at our institution. The degree of cervical myelopathy before and after surgery was assessed using the Japanese Orthopaedic Association (JOA) scoring system [5]. Briefly, this score comprises four items, including upper extremity motor function, lower extremity motor function, sensory function, and bladder function (Table 1). The JOA score is the sum of these items (I + II + III + IV in Table 1). The recovery rate of the JOA score was calculated to compare pre- and postoperative JOA scores as follows: Recovery rate (%) = (Postoperative score—Preoperative score) × 100/(17—Preoperative score). These clinical findings were recorded using electronic data capture (Claris FileMaker Pro 19; Claris International, Cupertino, CA) with security systems in place. The presence of dysphagia was defined as moderate or severe symptoms according to Bazaz score. The incidence of segmental paralysis (so-called C5 palsy), aspiration pneumonia, delirium, and deep venous thrombosis were recorded.

### 2.4. Radiologic Evaluations

Cervical sagittal alignment (C2–7 lordotic angle) was assessed using tangential lines drawn on the posterior edge of the C2 and C7 vertebral bodies on lateral radiographs acquired in a neutral standing position [10]. Preoperative center of the head—C7 sagittal vertical axis (C-SVA) [17] and T1 slope [18]—were also measured (Figure 1a). The fused segment angle (FSA) and fused segment height (FSH) were also determined. Briefly, FSA is the angle between lines drawn parallel to the cranial endplate of the cranial vertebrae of the fused segment and the caudal endplate of the caudal vertebrae of the fused segment, and FSH was determined as the mean value of the anterior and posterior vertebral body heights at the fused segments (Figure 1d,e) [19,20]. In the hybrid group, these parameters were independently calculated in the ACCF and ACDF segments. Additionally calculated were changes in both FSA (ΔFSA) and FSH (ΔFSH) between before and immediately after the operation, |ΔFSA| and |ΔFSH|. Graft migration was defined as subsidence >3 mm. Solid fusion was defined as the presence of continuous bone connecting the Luschka joints at the operated segments on X-ray. Radiologic measurements were performed by an independent assessor (M.H.). Formal analysis was performed by another independent assessor (T.H.). These two doctors are certified by the Japanese Society for Spine Surgery and Related Research to perform spine surgery. 

### 2.5. Statistical Analysis

Differences between the two groups were assessed using one-way analysis of variance, the Mann Whitney *U* test, or the Chi-square test. Multivariate logistic regression with a forward stepwise procedure was performed to identify key risk factors for postoperative implant migration (*p* < 0.1 for entry), with occurrence of graft migration as the objective variable and age, sex, and radiographic parameters as explanatory variables. All statistical analyses were carried out using SPSS for Windows (version 20.0; IBM Corp., Armonk, NY, USA). A *p*-value of less than 0.05 was considered statistically significant.

## 3. Results

### 3.1. Demographic Data and Clinical Outcomes

Patients (41 men, 12 women; follow-up rate, 100%) completed at least 2 years of follow-ups (Table 2). Of these, 30 patients were categorized into the ACCF group and 23 into the hybrid group. Mean preoperative/postoperative JOA scores were 11.9/15.0 points and 11.1/14.6 points, respectively. The average number of fused segments was 3.3 levels in the ACCF group and 3.5 levels in the hybrid group. Mean estimated blood loss and operative time were 437 mL and 6.5 h in the ACCF group and 197 mL and 6.0 h in the hybrid group, respectively. Duration of intensive care unit (ICU) stay and timing of extubation were respectively 2.8 days and 0.5 days in the ACCF group and 3.3 days and 0.9 days in the hybrid group. Duration of hospitalization was 24.2 days and 29.3 days, respectively. There were no significant differences in demographic and clinical characteristics between the two groups.

The incidence of perioperative complications was similar between the groups (Table 3). In the ACCF group, there was persistent dysphagia in four cases categorized as moderate by their Bazaz score (13.3%), aspiration pneumonitis in three (10%), delirium in two (6.7%), segmental paralysis in two (6.7%), and deep vein thrombosis in one (3.3%). In one case, dysphagia did not resolve until 2 months after the operation. However, other complications were resolved during the hospital stay. In the hybrid group, there was one case each (4.3%) of dyspnea caused by internal hematoma, aspiration pneumonitis, delirium, and segmental paralysis.

Revision surgery for implant failure was performed in five cases (16.7%) in the ACCF group but in none in the hybrid group (Table 3). However, additional corpectomy was required for one patient who developed segmental motor dysfunction in the hybrid group. The incidence of reoperation, especially due to strut dislodgement immediately postoperatively, was significantly higher in the ACCF group compared with the hybrid group (*p* = 0.04, Table 3).

### 3.2. Radiographic Outcomes

**Figure 2:**
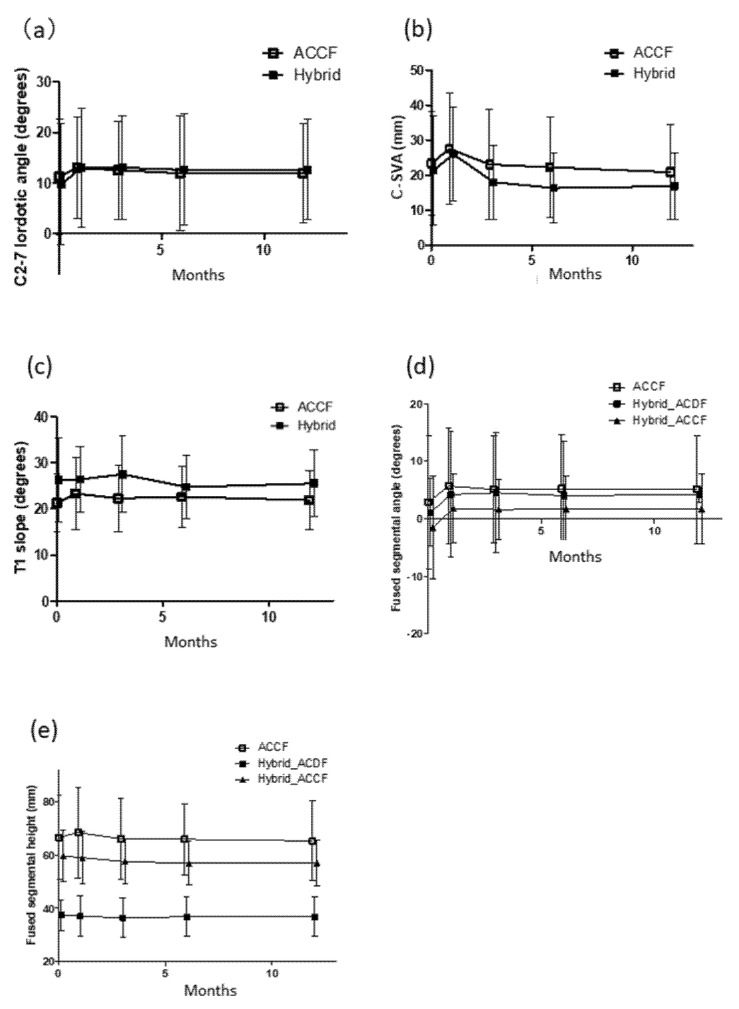
Radiographic measurement of (**a**) C2–7 angle; (**b**) C2–7 SVA; (**c**) T1 slope; (**d**) FSA; and (**e**) FSH in the ACCF and hybrid groups.

Chronological changes on radiographs were evaluated. Mean C2–7 angle increased immediately postoperatively and was maintained in both groups (Figure 2a). C-SVA was increased immediately postoperatively, but it had gradually decreased by 6 months postoperatively in both groups (Figure 2b). T1 slope did not change during the follow-up period (Figure 2c). In the ACCF group, mean FSA was increased immediately postoperatively and was maintained. However, there was a gradual decrease with a loss of 0.7 degrees 1 year after the operation. In the hybrid group, mean FSA was increased in segments treated with either ACDF or ACCF after surgery and was unchanged at the 1-year follow-up (Figure 2d).

In the ACCF group, mean FSH was increased by 2 mm immediately postoperatively. However, there was a 2-mm decrease at 1 year postoperatively. In the hybrid group, mean FSH was unchanged postoperatively, even compared with preoperatively (Figure 2e). Strut subsidence was observed in eight cases (26.7%) in the ACCF group and three cases (13.0%) in the hybrid group (Table 4). Among patients with strut subsidence, secondary surgery was required for early implant dislodgement in four cases in the ACCF group, but no secondary surgeries were required in the hybrid group. The fusion rate was significantly higher in the hybrid group than in the ACCF group (100% vs. 80%).

### 3.3. Association of Change in FSH Immediately Postoperatively with Strut Subsidence in the ACCF Group

**Figure 3:**
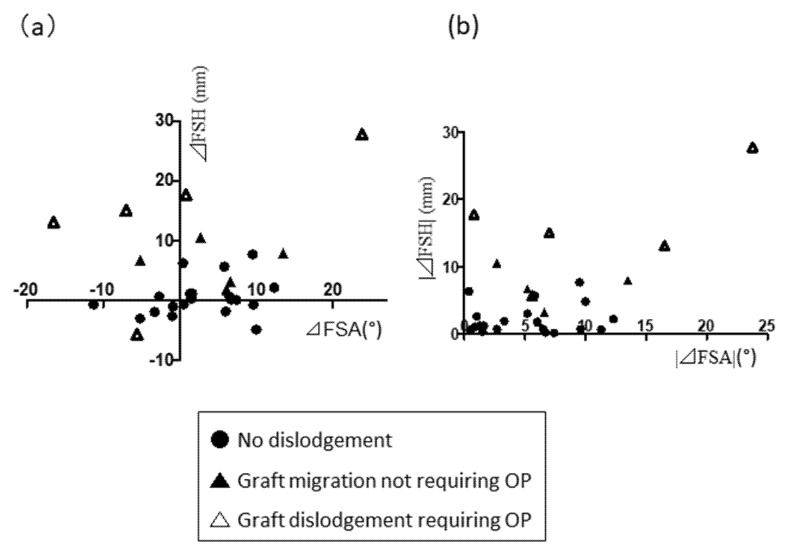
(**a**) Plot showing changes in FSA and FSH (ΔFSA and ΔFSH) and the incidence of graft subsidence; (**b**) Plot showing associations between absolute values of ΔFSA and ΔFSH and graft migration. A large |ΔFSH| is more closely linked with graft migration than |ΔFSA|.

To identify postoperative structural changes in the fused segments, associations between the incidence of graft dislodgement and parameters including FSA and FSH were evaluated in the ACCF group. Graft subsidence was more likely to occur in those with ΔFSH > 5 mm (Figure 3a). Of note, early strut dislodgement that required secondary surgery occurred in three of four cases with ΔFSH >10 mm. Stepwise logistic regression analysis demonstrated that only |ΔFSH| was a key risk factor for postoperative graft subsidence (odds ratio 1.328, 95% confidence interval 1.017–1.733; *p* = 0.04; Figure 3b).

## 4. Discussion

Various studies have discussed the superiority of certain surgical procedures for the treatment of patients with multilevel OPLL [5,6,8,9,21]. A meta-analysis revealed the anterior procedure provides more favorable results in terms of neurological recovery and postoperative cervical alignment [5]. Fujiyoshi et al. developed the K-line, which connects the midpoints of the spinal canal at C2 and C7 on neutral lateral radiographs, as a means of predicting poor clinical outcomes in patients with ossification of the posterior longitudinal ligament (OPLL) [22]. They classified OPLL patients into two groups, K-line (+) and K-line (−), and demonstrated that there was an insufficient posterior shift of the spinal cord and no neurologic improvement after posterior decompression surgery in patients in the K-line (−) group, in whom the anterior compression of OPLL exceeds the line. Notably, the classification is able to predict whether anterior compression of the spinal cord, which often impairs postoperative neural recovery [23], remains even after posterior decompression, and thus it is a very effective tool for deciding which surgical treatment—anterior or posterior—should be performed [8]. We also previously reported better outcomes after ACCF than after posterior procedures in patients who had severe OPLL with kyphotic alignment [9,21]. However, most comparative studies have shown that surgical complications were more frequent with anterior cervical surgery [6,21,24]. Of all complications after ACCF, airway obstruction and early graft migration are the most serious, often leading to emergency treatment and high reoperation rates [6,21,24]. For spine surgeons and neurosurgeons especially, it is important to recognize risk factors for predicting such perioperative complications.

This study also showed that the hybrid group had a relatively higher recovery rate in terms of JOA score (Table 1) and a lower incidence of complications (Table 2), although these differences did not reach significance. Additionally, no significant differences were found in terms of duration of ICU stay, the timing of postoperative extubation, hospital stay, or blood loss, although blood loss was certainly lower in the hybrid group. Fortunately, none of our patients needed a blood transfusion due to circulatory shock in this study. We speculate that corpectomy often violates the epidural venous plexus when decompressing the lateral portion of the spinal canal, thereby increasing the incidence of massive epidural bleeding in the ACCF procedure. Hospital stays were somewhat longer in the hybrid group than in the ACCF group, despite the lower complication rate in the hybrid group. Although there were more mechanical complications in the ACCF group, this finding suggests that the ACCF technique may be more invasive but may not affect the general condition of the patient as much as the hybrid procedure seems to.

Various studies have investigated the incidence of complications after anterior cervical surgery, which is reported to range from 11.3% to 64.3% [5,6,21,25,26,27]. Generally, it has been recognized that the hybrid procedure results in lower complication rates, ranging from 0% to 22.2%, and improved clinical outcomes compared with conventional ACCF, ranging from 6.2% to 43.6% [14,28,29]. In our study, the rate was 30.2% overall (ACCF, 40%; hybrid, 17.4%), relatively consistent with the results of previous studies. Of note, dysphagia occurred in four cases (13.3%) in the ACCF group, but did not occur in the hybrid group. A recent meta-analysis reviewed 38 studies and found an incidence of 16.8% after anterior cervical surgery [30]. Fortunately, the complication rate of dysphagia in this study was lower than previously reported. We speculate that because our institution is a high-volume center for cervical spine surgery, especially anterior cervical spine operation, the complication rate might be relatively low. Despite finding no significant difference between the two groups in our study, dysphagia caused by postoperative soft tissue swelling was likely lower in the hybrid group (0%) than in the ACCF group (13.3%). First, various surgical instruments, such as surgical osteotomes and retractors, were different between these two group because the patients categorized into the ACCF group underwent surgery in an earlier period than those in the hybrid group. Second, posterior cooling pads were placed on the anterior aspect of the neck immediately after surgery to prevent soft tissue swelling from 2012. In addition, we speculate that the superior laryngeal nerve and muscles related to swallowing might be less susceptible to mechanical damage in the hybrid group than in the ACCF group, likely because operative retractors can be loosened during decompression of the ossified lesions in the most compressed segment in the hybrid group, although they should be sufficiently expanded during decompression in the ACCF group. Because dysphagia can potentially develop even in patients treated with anterior hybrid fusion, it should be kept in mind postoperatively for both patients undergoing traditional ACCF and those undergoing hybrid fusion. Nevertheless, we believe that the hybrid technique could be a less invasive and more structurally stable strategy for treating severe OPLL compared with the conventional ACCF method.

It is known that multiple ACDF provides several advantages over multiple corpectomies [11]. ACDF is less invasive and is technically easier than ACCF. There is also less graft dislodgement, better correction of kyphotic deformity, and less need for postoperative immobilization because the screws are inserted into the preserved vertebral bodies [11]. However, there is some degree of difficulty involved in decompressing multilevel continuous lesions in the spinal canal and foramen using ACDF [8,20]. In patients with multilevel OPLL, corpectomy is often required to decompress the lesion entirely [20,21]. Vaccaro et al. reported that early graft dislodgement was seen in 9% of patients treated with 1-vertebra body corpectomy, and in as high as 50% of those receiving 2-vertebral body corpectomy [31]. Similarly, Okawa et al. reported that implant migration was observed in 30% of patients undergoing ACCF with average 3.8-level decompression [32]. The present study is the first to focus exclusively on severe OPLL affecting three or more levels and demonstrated that the incidence of reoperation for graft dislodgement was significantly higher in the ACCF group (16.7%) compared with the hybrid group (0%). In addition, mean FSH was decreased by 2 mm at 1 year after surgery in the ACCF group, whereas it was unchanged in the hybrid group (Table 3 and Figure 2e). Interestingly, mean FSH at 1 year returned to the preoperative value in the ACCF group. These findings suggest that the longer sized-graft bone could be applied to patients with ≥3 levels OPLL. As we previously reported, a postoperative increase in FSH can affect graft stability and lead to early implant migration [13]. The long lever arm created by long strut graft and fixation with four screws in the ACCF technique often stresses the screws and sometimes results in graft dislodgement [20]. A biomechanical study demonstrated that the hybrid technique was more effective for strengthening cervical stability and reconstructing sagittal alignment compared with the ACCF technique [33]. We also found that the fusion rate was significantly higher in the hybrid group at 1 year after the surgery. Taken together, our findings and previous findings indicate that the placement of two additional anchors in the hybrid technique can reduce stress on the proximal and distal screws and prevent loosening of the screws and graft dislodgement.

In this study, C2–7 lordotic angle and C-SVA were improved in both the ACCF and hybrid groups. Various studies have shown improvement and maintenance of cervical sagittal alignment after anterior cervical surgery [8,10,20,21]. This suggests that complete anterior decompression of ossified lesions may allow patients to assume a posture with more extension of the cervical spine. However, repeated micromotion can loosen the screws and could lead to severe reconstruction failure even while wearing a neck collar postoperatively. Particularly in cervical OPLL in patients with ankylosing spondylitis, reconstruction surgery with rigid fixation should be applied for the affected levels adjacent to the lesion. Interestingly, the prevalence of diffuse idiopathic skeletal hyperostosis (DISH) w shown to be approximately 45% in cervical OPLL patients [34,35]. Other observational studies have also demonstrated that multiple ossified lesions in the cervical spine often coexist with ossification of the spinal ligaments in the thoracolumbar spine [34,36,37,38]. In our study, of the four patients who underwent secondary surgery due to early implant failure in the ACCF group, three had DISH. Therefore, reconstruction surgery with rigid fixation and adequate structural alignments, such as hybrid fusion or anterior pedicle screw fixation [39], should be performed for cervical OPLL in patients with ankylosing thoracic spondylitis.

This study has several limitations. First, this was a retrospective study with a relatively small number of subjects and heterogeneity in terms of the implants used. Second, the degree of preservation of the cephalic and caudal endplates was not consistent in our series, although it was preserved as much as possible. Third, we could not assess factors associated with the location of the graft or screw angle and length. Fourth, these two techniques were performed by several independent surgeons. Fifth, the period was different between the two methods. Nevertheless, despite these limitations, our study highlights the fact that FSH should not be increased extremely after graft placement and plating should be carried out to prevent postoperative graft dislodgement in ACCF. Additionally, the hybrid fusion procedure is recommended for multilevel cervical OPLL in patients with ankylosing spondylitis to achieve sufficient stability of fused segments.

## 5. Conclusions

This study is the first to compare conventional ACCF and the hybrid fusion technique focusing exclusively on patients with severe OPLL involving three or more levels. Although both procedures can achieve satisfactory neurologic outcomes in patients with multilevel OPLL, hybrid fusion was superior to the conventional ACCF technique in terms of fusion rate and perioperative graft stability.

## Figures and Tables

**Table 1:** Scoring system for cervical myelopathy (JOA score).

I Upper extremity motor function		
	0:	Unable to feed oneself with any tableware including chopsticks, spoon, or fork, and/or unable to fasten buttons of any size
	1:	Can manage to feed oneself with spoon and/or fork but not chopsticks
	2:	Either chopsticks feeding or writing is possible but not practical, and/or large buttons can be fastened
	3:	Either chopsticks feeding or writing is clumsy but practical, and/or cuff buttons can be fastened
	4:	Normal
II Lower extremity motor function		
	0:	Unable to stand up and walk by any means
	0.5:	Able to stand up but unable to walk
	1:	Unable to walk without a cane or other support on level ground
	1.5:	Able to walk without support but with a clumsy gait
	2:	Walks independently on level ground but needs support on stairs
	2.5:	Walks independently when going upstairs, but needs support when going downstairs
	3:	Capable of walking fast but clumsily
	4:	Normal
III Sensory function		
A. Upper extremity		
	0:	Complete loss of touch and pain sensation
	0.5:	50% or below of normal sensation and/or severe pain or numbness
	1:	Over 60% of normal sensation and/or moderate pain or numbness
	1.5:	Subjective numbness of a slight degree without any objective sensory deficit
	2:	Normal
B. Lower extremity		
		Same as A
C. Trunk		
		Same as A
IV Bladder function		
	0:	Urinary retention and/or incontinence
	1:	Sensory of retention, dribbling, thin stream and/or incomplete continence
	2:	Urinary retardation and/or pollakiuria
	3:	Normal

**Table 2:** Demographic and clinical characteristics in the ACCF and hybrid groups.

	ACCF Group (*n* = 30)	Hybrid Group (*n* = 23)	*p*
Age (years)	61.7 ± 9.1	62.9 ± 10.5	0.71
Male:Female	24:6	17:6	0.74
Diabetes mellitus (%)	10 (33.3)	6 (26.1)	0.57
History of smoking (%)	12 (40)	7 (30.4)	0.47
Preoperative JOA score (points)	11.9 ± 2.1	11.1 ± 3.8	0.39
Postoperative JOA score (points)	15.0 ± 1.4	14.6 ± 2.3	0.50
Recovery rate of JOA score (%)	56.3 ± 32.1	70.4 ± 27.0	0.12
No. of fused segments	3.3 ± 0.6	3.5 ± 0.7	0.33
Graft type in ACCF part	Artificial bone 20 Fibular graft 6 Iliac graft 4	Artificial bone 20 Fibular graft 3	0.28
Graft type in ACDF part	-	Artificial bone 20 fusion cage 3	-
Estimated blood loss (mL)	437 ± 778	197 ± 151	0.20
Operating time (h)	6.5 ± 2.4	6.0 ± 2.4	0.40
Duration of ICU stay (days)	2.8 ± 1.4	3.3 ± 2.6	0.39
Time to postoperative extubation (days)	0.5 ± 1.1	0.9 ± 2.6	0.55
Hospital stay (days)	24.2 ± 10.7	29.3 ± 12.3	0.55

Data are shown as the mean ± standard deviation. ACCF, anterior cervical corpectomy with fusion; JOA, Japanese Orthopaedic Association; ICU, intensive care unit.

**Table 3:** Perioperative complications in the ACCF and hybrid groups.

	ACCF Group	Hybrid Group	*p*
	(*n* = 30)	(*n* = 23)	
Complications, *n* (%)			
Total	12 (40%)	4 (17.4%)	0.08
Dysphagia	4 (13.3%)	0 (0%)	0.07
Aspiration pneumonitis	3 (10%)	1 (4.3%)	0.44
Delirium	2 (6.7%)	1 (4.3%)	0.72
Segmental paralysis	2 (6.7%)	1 (4.3%)	0.72
DVT	1 (3.3%)	0 (0%)	0.37
Dyspnea (internal hematoma)	0 (0%)	1 (4.3%)	0.24
Revision surgery, *n* (%)			
Total	5 (16.7%)	1 (5.3%)	0.16
Graft dislodgement	5 (16.7%)	0 (0%)	0.04 *
Segmental paralysis	0 (0%)	1 (5.3%)	0.24

Data are shown as the mean ± standard deviation. ACCF, anterior cervical corpectomy with fusion; DVT, Deep vein thrombosis; * Statistically significant, *p* < 0.05.

**Table 4:** Radiologic outcomes in the ACCF and hybrid groups.

		ACCF Group (*n* = 30)	Hybrid Group (*n* = 23)		*p*	
C2–7 angle (°)	Preoperative	11.2 ± 11.4	9.7 ± 12.0		0.76	
	Immediate postoperative	13.0 ± 10.1	13.0 ± 11.7		0.94	
	1 year	11.9 ± 9.8	12.6 ± 9.9		0.66	
C-SVA (mm)	Preoperative	23.4 ± 14.9	21.4 ± 15.5		0.97	
	Immediate postoperative	27.6 ± 16.0	26.0 ± 13.5		0.59	
	1 year	20.9 ± 13.5	16.9 ± 9.5		0.39	
T1 slope (°)	Preoperative	21.2 ± 6.3	26.2 ± 9.1		0.77	
	Immediate postoperative	23.3 ± 7.8	26.4 ± 7.2		0.27	
	1 year	21.9 ± 6.4	25.5 ± 7.3		0.61	
		ACCF	ACDF part	ACCF part	Overall	
FSA (°)	Preoperative	2.9 ± 11.6	1.1 ± 5.9	−1.6 ± 8.9	1.9 ± 11.3	0.77 ^#^
	Immediate postoperative	5.7 ± 10.0	4.2 ± 10.9	1.8 ± 6.0	4.7 ± 10.8	0.27 ^#^
	1 year	5.1 ± 9.4	4.2 ± 1.3	1.7 ± 6.0	4.3 ± 10.4	0.36 ^#^
FSH (mm)	Preoperative	66.6 ± 15.7	37.4 ± 5.8	59.6 ± 9.6	69.4± 16.3	0.66 ^#^
	Immediate postoperative	68.4 ± 17.1	37.0 ± 7.7	59.0 ±9.9	69.1 ± 15.4	0.54 ^#^
	1 year	66.4 ± 15.0	36.8 ± 7.4	58.4 ± 8.6	68.8 ± 15.1	0.37 ^#^
ΔC2–7 angle (°)		1.8 ± 8.2	3.3 ± 8.2		0.53	
ΔFSA (°)		2.8 ± 7.9	2.6 ± 8.1	2.8 ± 7.5	2.8 ± 8.4	0.96 ^#^
ΔFSH (mm)		3.2 ± 7.1 *	−0.4 ± 3.0	−0.6 ± 3.5	1.4 ± 7.7	0.23 ^#^
Graft subsidence (cases)		8 (26.7%)	3 (13.0%)		0.22	
Fusion rate		80%	100% *		0.02 *	

Data are shown as the mean ± standard deviation. ACCF, anterior cervical corpectomy with fusion; ACDF, anterior cervical discectomy with fusion; C-SVA, cervical-sagittal vertical axis; FSA, fused segmental angle; FSH, fused segmental height. * Statistically significant, *p* < 0.05. ^#^ Compared between the ACCF group and all patients in the hybrid group.

## Data Availability

Detailed data are available on request from corresponding author.
